# Associations among stressors across the lifespan, disability, and relapses in adults with multiple sclerosis

**DOI:** 10.1002/brb3.3073

**Published:** 2023-05-21

**Authors:** Carri S. Polick, Robert Ploutz‐Snyder, Tiffany J. Braley, Cathleen M. Connell, Sarah A. Stoddard

**Affiliations:** ^1^ School of Nursing Duke University Durham North Carolina USA; ^2^ School of Nursing University of Michigan Ann Arbor Michigan USA; ^3^ Applied Biostatistics Laboratory University of Michigan Ann Arbor Michigan USA; ^4^ Division of Multiple Sclerosis & Neuroimmunology Department of Neurology Michigan Medicine Ann Arbor Michigan USA; ^5^ School of Public Health University of Michigan Ann Arbor Michigan USA

**Keywords:** disability, multiple sclerosis, relapses, traumatic stress

## Abstract

**Introduction:**

Stress and adversity during childhood, adolescence, and adulthood could impact the present and future health and well‐being of people with multiple sclerosis (PwMS); however, a lifespan approach and nuanced stressor data are scarce in this nascent area of research. Our aim was to examine relationships among comprehensively measured lifetime stressors and two self‐reported MS outcomes: (1) disability and (2) relapse burden changes since COVID‐19 onset.

**Methods:**

Cross‐sectional data were collected from a nationally distributed survey of U.S.‐based adults with MS. Hierarchical block regressions were used to sequentially evaluate contributions to both outcomes independently. Likelihood ratio (LR) tests and Akaike information criterion (AIC) were used to evaluate additional predictive variance and model fit.

**Results:**

A total of 713 participants informed either outcome. Most respondents (84%) were female, 79% had relapsing remitting multiple sclerosis (MS), and mean (*SD*) age was 49 (12.7) years. Childhood (*R* ^2^ = .261, *p* < .001; AIC = 1063, LR *p* < .05) and adulthood stressors (*R* ^2^ = .2725, *p* < .001, AIC = 1051, LR *p* < .001) contributed significantly to disability, above and beyond prior nested models. Only adulthood stressors (*R* ^2^ = .0534, *p* < .001; AIC = 1572, LR *p* < .01) significantly contributed above the nested model for relapse burden changes since COVID‐19.

**Conclusions:**

Stressors across the lifespan are commonly reported in PwMS and could contribute to disease burden. Incorporating this perspective into the “lived experience with MS” could facilitate personalized health care by addressing key stress‐related exposures and inform intervention research to improve well‐being.

## Graphical abstract

The effect of stressors across the lifespan on Multiple Sclerosis is an under‐researched area. We found that both childhood and adulthood stressors contributed to disability; while only adult stressors contributed to relapse burden since Covid‐19 onset. This study highlights the importance of comprehensively addressing lifetime stressors

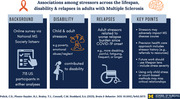

## 1 INTRODUCTION

Multiple sclerosis (MS), an immune‐mediated inflammatory disease, is the leading cause of nontraumatic disability in young adults and is the second highest with respect to direct medical expenses associated with a chronic illness in the United States (Hartung, 2021). The potential link between childhood stress and MS disease burden has been a burgeoning area of research; however, relationships between stress and MS pathogenesis and progression remain unclear due to mixed and insufficient evidence (Polick et al., 2022). For example, there are two available studies regarding disability. Spitzer et al. (2012) did not find a relationship between childhood trauma and adult disability in people with multiple sclerosis (PwMS). Although Horton et al. (2022) did initially identify higher odds of walking aid dependence in PwMS who experienced four or more adverse childhood experiences (ACEs), after adjusting for multiple testing comparisons, the significance of this finding became nonsignificant. Childhood physical and sexual abuse have been significantly associated with higher relapse rates in PwMS, but these relationships have only been explored in one study (Spitzer et al., 2012).

Limitations to the measurement of ACEs, which is count based and lacks nuance to assess severity, and to study designs (e.g., limited sample size, covariates) likely contribute to these conflicting results. For example, none of the studies in a recent systematic review (*n* = 12) controlled for disease‐modifying therapies, and only two studies accounted for smoking/substance use (Goodwin & Stein, 2004; Riise et al., 2011), which can both alter disability and relapses (Polick et al., 2022). Yet, evidence suggests that traumatic childhood stressors including abuse, neglect, and household dysfunction could alter brain development, cause aberrant inflammatory responses, increase the perception of adult stress, and lead to maladaptive lifestyle behaviors that increase the risk of MS or disease progression (e.g., smoking, obesity) (Albott et al., 2018; Centers for Disease Control & Prevention, 2022; McEwen, 2017). Acute stress responses can progress to chronic stress, through amygdala‐related pathways that sensitize to threat detection over the lifespan (McEwen, 2017). Thus, to inform efforts that address disease burden and treatment strategy, it is important to expand beyond an exclusive focus on childhood to incorporate adult stressors.

Few studies have used a lifespan approach to ascertain the cumulative effect of stressors on MS, and to the best of our knowledge, none have focused on physical symptoms of disability or relapse burden (Polick et al., 2022). The purpose of this study is to characterize relationships among comprehensively measured lifetime stressor exposures (e.g., child, adult, severity, expanded criteria), and two clinically relevant MS outcomes: disability severity and relapse burden changes since COVID‐19 onset.

## 2 METHODS

Self‐report data were collected by emailing a REDCap survey link to the U.S. National MS Society listserv of nearly 80,000 PwMS on October 18, 2021; the link remained open until November 4th. Eligibility criteria included English reading adults who report being officially diagnosed with MS. This study and report are guided by STROBE criteria (Von Elm et al., 2014).

Stressors were measured with the Stress and Adversity Inventory (STRAIN), which captured the cumulative count and severity (i.e., 1–5 Likert scale) of 55 stressors over the lifespan (Slavich & Shields, 2018). Examples include physical/sexual abuse, neglect, poverty, divorce, neighborhood violence, and witnessing domestic abuse. Stressors occurring before age 18 were classified as childhood stressors. The STRAIN was validated with an autoimmune sample and has shown good test–retest reliability over time (Slavich & Shields, 2018).

Disability severity was measured with the Patient Determined Disease Steps (PDDS), a one‐item ordinal 0–8 scale commonly converted into three categories of mild (0–1), moderate (2–4), and severe (5–8) disability (Learmonth et al., 2013). The PDDS has been validated for PwMS and strongly correlates with clinical findings (Learmonth et al., 2013).

Due to the potential of COVID‐19 stress confounding relapse rates, we instead measured how relapse burden changed since COVID‐19 onset (March 2020). Participants could report more/less pain, fatigue, disability, frequency, and length. Responses were categorized into an ordinal scale from (0) *No relapse*, which was most ideal, to (1) *Lighter burden* (e.g., less disabling, shorter), (2) *No change*, or (3) *Worse burden* (e.g., more painful).

Hierarchical block ordinal logistic regression modeling in STATA was used to sequentially assess baseline demographic and covariate contributions to each outcome. Covariates shown in Tables 2 and 3 were tailored per each outcome, encompassing known risk factors (e.g., smoking) and factors hypothesized to be relevant (e.g., seasonality). Next, we compared model fit of the base model to our second model, which included the base predictors plus childhood stressor predictors to determine if these early stressors contribute to MS outcomes over and above the base model. Finally, we added a third set of predictors evaluating the contributions of adult stressors to determine if they contributed additional predictive variance overall. Each successive model was compared to the prior model using likelihood ratio (LR) testing, and we report Akaike information criterion (AIC) as an index of relative model fit, with a lower number indicating better fit. Analyses were powered to detect 4% changes between models. Due to case‐wise deletion, only participants with complete cases per outcome were included. It is important to note that this hierarchical block strategy assumes collinearity between similar stressors clustered together in model blocks to represent a latent concept (e.g., childhood stress), and thus may make individual stressor variables appear unremarkable. Therefore, interpretation is conducted at the model level to provide foundational evidence of relationships in this developing area of research.

## 3 RESULTS

Of the 713 participants included in either of the final analytical models, most were female (*n* = 597, 84%), White (*n* = 415, 88%), and with Relapsing Remitting MS (*n* = 559, 79%), aligning with the conventional MS research population (National MS Society, 2023) (Table 1). Most were on a second‐line disease‐modifying therapy (*n* = 308, 44%), had mild disability (*n* = 365, 52%), and on average experienced 2.6 (*SD* = 1.96) childhood stressors with a severity of 9.8 (*SD* = 8.8) and 23.6 (*SD* = 14) adulthood stressors with a severity of 55.4 (*SD* = 30.8).

**TABLE 1:** Sample characteristics.

Age, *M* (*SD*) (*n* = 713)	49 (12.7) range: 21–85
Gender, *n* (%) (*n* = 713)	
Female	597 (84%)
Male	100 (14%)
Transgender, nonbinary, gender nonconforming, or other	15 (2%)
Race/ethnicity, *n* (%) (*n* = 473)	
White	415 (88%)
Black	23 (5%)
Latinx	2 (< 1%)
Asian	4 (< 1%)
American Indian or Alaska Native	2 (< 1%)
Native Hawaiian or Pacific Islander	1 (< 1%)
Biracial or mixed	24 (5%)
Education, *n* (%) (*n* = 713)	
High school equivalency, or below	36 (5%)
Associates degree or some college	167 (23%)
Bachelor's degree	259 (36%)
Master's degree or above	251 (35%)
Smoking status, *n* (%) (*n* = 709)	
Never smoker	465 (66%)
Former smoker	198 (28%)
Current or social smoker	46 (6%)
Birth season, *n* (%) (*n* = 710)	
Spring	187 (26%)
Summer	175 (25%)
Fall	178 (25%)
Winter	170 (24%)
MS phenotype, *n* (%) (*n* = 713)	
Relapsing remitting MS	559 (79%)
Primary progressive MS	35 (5%)
Secondary progressive MS	87 (12%)
Progressive relapsing MS	9 (1%)
Unsure	23 (3%)
Length of time since MS onset, *M* (*SD*) (*n* = 713)	18 (12) range: 0–59
Disease‐modifying therapy, *n* (%) (*n* = 709)	
None	129 (18%)
First line	272 (38%)
Second line	308 (44%)
Stressors, *M* (*SD*) (*n* = 713)	
Childhood count	2.6 (1.96)
Childhood severity	9.8 (8.8)
Adult count	23.6 (14)
Adult severity	55.3 (30.8)
Outcome variables	
Disability (PDDS), *n* (%) (*n* = 706)	
Mild	365 (52%)
Moderate	236 (33%)
Severe	105 (15%)
Relapse change since COVID‐19 onset (*n* = 675)	
No relapse	198 (29%)
Improved	26 (4%)
Stayed the same	242 (36%)
Worsened	209 (31%)

Abbreviations: MS, multiple sclerosis; PDDS, Patient Determined Disease Steps.

### 3.1 Disability

The base model including demographic and MS history predictors contributed significantly to estimates of disability level (*R* ^2^ = .256, *p* < .001; model AIC = 1066) (Table 2). The childhood stressors in model 2 contributed a significant amount of variance over the base model (*R* ^2^ = .261, *p* < .001; AIC = 1063, LR *p* < .05). The adult stressor predictors in model 3 also contributed significantly over the prior nested models (*R* ^2^ = .2725, *p* < .001; AIC = 1051, LR *p* < .001), meaning all predictor blocks (baseline, childhood stressors, adult stressors) significantly related to disability, and collectively contribute 27.3% of variance to this outcome.

**TABLE 2:** Final analytic hierarchical model of disability using ordinal logistic regression (*n* = 695).

					Overall model statistics		
	OR	*SE*	95% CI	*p*	*R* ^2^	AIC	LR test
Covariates in base model				**<.0001**	.2560	1066	Base
Age	1.05	0.01	1.03–1.07	**<.001**			
Gender (ref. female)							
Male	1.10	0.27	0.67–1.78	.72			
Transgender, nonbinary, gender nonconforming	0.47	0.29	0.14–1.58	.22			
Education (ref. ≤high school)							
Associates degree or some college	0.82	0.33	0.37–1.78	.61			
Bachelor's degree	0.52	0.21	0.24–1.14	.11			
Master's degree or above	0.33	0.13	0.15–0.72	**<.01**			
Smoking status (ref. never smoker)							
Former smoker	1.08	0.21	0.74–1.59	.70			
Current or social smoker	1.83	0.64	0.93–3.64	.08			
Birth season (ref. Spring)							
Summer	0.61	0.14	0.39–0.97	**<.04**			
Fall	0.67	0.16	0.43–1.06	.09			
Winter	0.61	0.14	0.38–0.97	**<.04**			
Time since MS onset (years)	1.02	0.01	1.00–1.04	**<.05**			
MS phenotype (ref. RRMS)							
Primary progressive MS	23.07	9.76	10.07–52.84	**<.001**			
Secondary progressive MS	15.57	4.52	8.82–27.50	**<.001**			
Progressive relapsing MS	31.26	24.41	6.77–144.41	**<.001**			
Unsure	1.47	0.73	0.55–3.91	.44			
Disease‐modifying therapy (ref. None)							
First line	0.70	0.18	0.43–1.14	.15			
Second line	1.55	0.38	0.96–2.52	.08			
Childhood stressors added for model 2				**<.0001**	.2611	1063	**.03**
Child stressor count	1.27	0.20	0.93–1.73	.13			
Child stressor severity	0.95	0.04	0.88–1.02	.14			
Adult stressors added for model 3				**<.0001**	.2725	1051	.**0004**
Adult stressor count	1.01	0.02	0.98–1.05	.40			
Adult stressor severity	1.01	0.01	1.00–1.03	.25			

Abbreviations: AIC, Akaike information criterion; CI, confidence interval; LR, likelihood ratio; MS, multiple sclerosis; OR, odds ratio; RRMS, relapsing remitting MS.

In addition to the overall blocks significantly contributing to disability level, individual predictors were also significant in the final model. Participants born in the Spring had 39% higher odds of disability compared to those born in the Summer (OR = 0.61, *p* < .05) and Winter (OR = 0.61, *p* < .05), which is consistent with how birth season influences the risk for MS at higher latitudes (Ismailova et al., 2019).

#### 3.1 Relapse burden change since COVID‐19 onset (March 2020)

The base model of predictors estimated the relapse burden since the onset of COVID‐19 (*R* ^2^ = .0455, *p* < .001; model AIC = 1581) (Table 3). The childhood stressors in model 2 increased the variance over the base model (*R* ^2^ = .048; AIC = 1580), and it was an overall significant model (*p* < .001); however, LR testing revealed that it was not significant compared to the previous nested base model (LR *p* = .08). The adult stressors in model 3 contributed significantly more information over the prior nested model (*R* ^2^ = .0534, *p* < .001; AIC = 1572, LR *p* < .01); therefore, only the base demographics/covariates and adult stressor blocks of predictors remained in the final analytic model.

**TABLE 3:** Final analytic hierarchical model of relapse burden change since COVID‐19 onset, using ordinal logistic regression (*n* = 668).

					Overall model statistics		
	OR	*SE*	CI	*p*	*R* ^2^	AIC	LR test
Covariates in base model				**<.0001**	.0455	1581	Base
Age	0.98	0.01	0.97–0.99	**<.001**			
Gender (ref. female)							
Male	0.92	0.20	0.61–1.40	.70			
Transgender, nonbinary, gender nonconforming	0.68	0.36	0.24–1.92	.47			
Education (ref. ≤high school)							
Associates degree or come college	0.41	0.16	0.19–0.87	**.02**			
Bachelor's degree	0.51	0.19	0.24–1.07	.08			
Master's degree or above	0.43	0.17	0.20–0.91	**.03**			
Smoking status (ref. neve smoker)							
Former smoker	0.96	0.17	0.68–1.35	.80			
Current or social smoker	0.86	0.27	0.46–1.59	.63			
MS phenotype (ref. relapsing remitting MS)							
Primary progressive MS	0.14	0.06	0.06–0.34	**<.001**			
Secondary progressive MS	0.64	0.15	0.40–1.02	.06			
Progressive relapsing MS	0.86	0.62	0.21–3.50	.83			
Unsure	1.47	0.65	0.62–3.47	.39			
Disease‐modifying therapy (ref. none)							
First line	1.21	0.27	0.79–1.87	.38			
Second line	1.23	0.27	0.80–1.89	.36			
Adult stressors added for final model				**<.0001**	.0534	1572	**<.002**
Adult stressor count	1.00	0.01	0.97–1.02	.77			
Adult stressor severity	1.01	0.01	1.00–1.02	.065			

Abbreviations: AIC, Akaike information criterion; CI, confidence interval; LR, likelihood ratio; MS, multiple sclerosis; OR, odds ratio.

Regarding individual predictors, childhood stress severity was significantly associated with increased relapse burden (*b* = .07, *p* = .03) in model 2; however, this lost significance when adding the adult stressor block for model 3 suggesting shared variance. With each year that the age of participants increased, there was a 2% reduction in relapse burden.

## 4 DISCUSSION

Our findings reveal associations among (1) childhood and adulthood stressors and MS disability level, and (2) adult stressors and MS relapse burden change since COVID‐19. Contrary to previous studies (Horton et al., 2022; Spitzer et al., 2012), our findings support associations between childhood stressors and disability, possibly due to more comprehensive measurement (e.g., severity, more stressors). Studies that use the ACE tool, or other measures that strictly capture stressor count, may underestimate results compared to studies that capture more nuanced stressor information like severity, duration, or frequency. In this study, childhood stressor severity was initially a significant predictor of relapse burden, but lost significance when further accounting for adult stressors, highlighting the importance of using a lifespan approach. Studies that lack adult stressor experiences may be missing important predictive information and may overestimate relationships among childhood stressors and health outcomes and/or miss the contributions of adult stress altogether.

Stressor timing may impact MS outcomes differently, based on mechanisms and competing factors. For example, disability could be in part triggered by longer term maladaptive coping behaviors such as earlier smoking or habits that contribute to obesity secondary to childhood stressors. However, adult stressors may be more likely to trigger aberrant immune responses (McEwen, 2017).

Our study found that birth seasonality impacted disability. Seasonality is more conventionally included in analyses of MS incidence risk and, until now, has not been included in studies assessing childhood or lifetime stressors and MS symptoms.

Variation in vitamin D absorption and its effects on immunomodulation during fetal immune system development in utero could help explain the relationship between birth season and adult MS disability (Palaniswamy et al., 2015). Future research should consider using pertinent MS covariates such as birth seasonality and smoking while investigating factors of disability.

Future work with a lifespan approach is needed to better understand and inform symptom management for PwMS, especially work that expands upon the few studies investigating childhood adversity and pain (MacDonald et al., 2021), fatigue (Pust et al., 2020), and psychiatric comorbidity (Wan et al., 2022). Further, ACE research has been growing in many adjacent areas of symptom severity such as functional neurological disorder (Paredes‐Echeverri et al., 2023), Tourette's syndrome (Yang et al., 2022), frequent headaches (Anto et al., 2021), and Parkinson's disease (Subramanian et al., 2023). A lifespan approach may similarly increase nuance in these areas as well as MS.

### 4.1 Limitations

Limitations include cross‐sectional and subjective self‐report data, which could be subject to recall bias, from a small portion of the 80,000 PwMS on the National MS Society listserv. Although a convenience sample, this approach had the advantage of reaching a large national U.S. sample of PwMS, as previous work has been conducted with smaller and/or clinic‐based samples, so this is a noted departure. This approach may also introduce selection bias toward people who have, and can navigate, internet access, which may not be inclusive of those with lower functionality or socioeconomic status.

## 5 CONCLUSIONS

This study highlights how stressors across the lifespan can help conceptualize when stressors matter most, and how nuanced measurement (e.g., count vs. severity) may play critical roles for MS disability level and relapse burden. These findings could help inform research design and analysis in this growing field to mitigate or prevent adverse outcomes associated with stressors across the lifespan. This work may also inform clinical conversations regarding stress reduction techniques for PwMS, and may facilitate quicker referrals to resources that may aid these efforts such as mental health, coping, or substance use support, thus providing more personalized care for PwMS.

## CONFLICT OF INTEREST STATEMENT

The authors declare no conflicts of interest.

### PEER REVIEW

The peer review history for this article is available at https://publons.com/publon/10.1002/brb3.3073.

## Data Availability

Data available on request due to privacy/ethical restrictions.
